# Supplementary material for: Are carbon emissions trading and green financial instruments synergistic? -Comprehensive quantitative research based on content analysis
Source: PLoS One. 2024 Mar 7;19(3):e0298601. doi: 10.1371/journal.pone.0298601 (PMC10919601; doi:10.1371/journal.pone.0298601)
Supplement: S1 Data — (ZIP) [file pone.0298601.s001.zip › Data/地级市Z_F工作报告.zip!/╣π╢1⁄2/├╖╓▌╩╨/└┤╫╘╣1⁄2╓┌║┼í╛┬φ┐╦╩2╛▌═°í┐_2018.docx]

各位代表：

现在，我代表梅州市人民政府向大会作报告，请予审议。并请政协各位委员和其他列席人员提出意见。

2017年工作回顾

2017年，是梅州加快改善发展条件、积极培育内生发展动力，经济社会实现平稳发展的一年。一年来，市政府在市委的正确领导下，在市人大和市政协的监督支持下，深入学习贯彻党的十九大精神、习近平总书记系列重要讲话以及对广东工作的重要指示批示精神，深化运用“两大振兴政策”，聚焦产业和项目建设，努力构建“一区两带六组团”发展新格局。全市生产总值1125.82亿元，增长6.8%；一般公共预算收入108.55亿元，增长5.0%；固定资产投资806.77亿元，增长24.0%、居全省第二。

一、打基础补短板有新进展。抓住国家扩大有效投资窗口期，致力于交通、水利、城市公共服务等基础设施项目建设，269个省市重点项目完成投资681亿元，占年度投资计划113%。民间投资活跃，完成投资484.36亿元，增长15.9%。综合交通条件持续改善，兴华高速建成通车，梅平、大潮（含大漳支线）等5条高速顺利推进，全市在建高速公路总数和建设里程为历年之最；梅汕高铁全线无障碍施工，双龙高铁前期工作取得突破性进展；300公里国省道改造项目开工建设，完成500公里县乡公路安全生命防护工程。全面实施河长制，完成322.5公里山区中小河流治理、8宗小流域综合治理。韩江高陂水利枢纽工程超额完成投资计划，梅州抽水蓄能电站稳妥推进。积极争取原中央苏区政策扶持资金，全面推进医疗卫生项目建设，加快市第三人民医院整体搬迁、市人民医院和市中医医院升级改造，改扩建27家县级医院，完成全市所有村卫生站改造。统筹推进农村配电网、光纤入户和公共区域WIFI等信息基础设施建设，完成农村无线高速局域网建设，城乡公共服务水平进一步提升。

二、实体经济发展有新成效。坚持创新驱动，认真落实省“实体经济十条”，切实加大产业培育扶持力度，产业结构渐趋优化。工业稳中向好，全市净增规上工业企业26家，规上工业增加值204.41亿元、增长1.4%。大力推进广东梅兴华丰产业集聚带建设，梅畲快速干线、广铁物流园、环保基础设施等重点项目加速推进，国家级经济技术开发区、国家级高新区、综合保税区申报创建工作有序推进。深化产业共建，新落地亿元以上项目26个，承接珠三角产业梯度转移项目51个；广梅园“园中园”建设取得新进展，王老吉原液提取基地、万宝家电生产基地正式动工。全市省级产业园区实现规上工业增加值75.73亿元，增长13.1%。发展全域旅游，梅江韩江绿色健康文化旅游产业带建设初见成效。25个旅游特色镇、67个旅游示范村和121个文化旅游产业项目加快建设，丰顺公式隍潮客小镇入选第二批全国特色小镇，五华横陂足球小镇入选首批全国运动休闲特色小镇。预计全市旅游接待总人数、总收入分别增长17%、18%。深化运用“互联网+”模式，完成梅州市跨境电商分拣清关中心主体项目建设，搭建农村电商、农邮合作发展平台，拓宽便民利民、助农增收渠道。社会消费品零售总额677.63亿元，增长9.3%。金融服务经济发展力度加大，金融机构存贷比提高3.24个百分点。新增8家新三板挂牌企业；梅州客商银行挂牌开业。大力推动大众创业、万众创新，认定25个省市小微企业创新创业示范基地，新登记各类市场主体增长22.4%；新增2家科技企业孵化器、7家众创空间，新认定72家高新技术企业，主营业务收入5亿元以上的工业企业实现研发机构全覆盖。丰顺建成全国首个出口电声产品质量安全示范区，博敏电子获省政府质量奖。外向型经济稳步发展，外贸进出口总额132.7亿元。成功举办第五届世界客商大会，搭建招商引资、开放发展新平台，全年共引进亿元以上项目111个。坚持差异化、特色化发展县域主导产业，荷树园电厂获省指标奖励，超华科技高精度电子铜箔工程一期项目试产，塔牌600万吨熟料水泥生产线一期项目投产；粤台（梅州）自行车产业园动工建设，其中凯达共享单车整车生产项目实现当年投产。县域经济完成地区生产总值685.64亿元，增长6.0%。

三、城乡人居环境有新面貌。加快城乡一体化建设，逐步拉开城市架构，不断完善功能，城市更具魅力。《梅州市城市总体规划（2015-2030年）》获省批复，梅县区城市规划区范围内的规划管理权限依法上收。嘉应新区起步区地下综合管廊和市政路网工程加快推进，世界客商中心、马鞍山公园、芹洋湿地公园等项目建成使用。引进和建成一批产城融合项目，中心城区吸引人气、集聚商气的能力不断增强。推进城市共治共管，率先在全省实行城市管理执法体制改革，城市综合管理执法权限下放梅江区；数字城管全面试运行。实施城市“双修”工程，城区绿化美化亮化水平不断提高。深入开展城乡人居环境整治，实现省卫生城市、卫生县城全覆盖。扎实推进34座污水处理厂、330公里污水管网和1580座农村污水处理设施建设，县城生活垃圾无害化处理率达100%，农村生活垃圾有效处理率达90%以上。坚持以点带面，中心镇“六个一”建设有序推进。加强森林资源管护，森林覆盖率达75.06%、居全省首位。综合防治大气、水、土壤污染，2017年梅州城市空气质量优良率居全省第一，饮用水源地水质达标率100%。完成节能减排示范市节能减排任务。韩江入选全国2017年度“最美家乡河”。

四、农业农村发展水平有新提高。加快发展现代农业，打响“世界客都·长寿之乡”品牌，推行集约化、产业化的生产与销售模式，带动农民增收。全市农业增加值212.35亿元，增长3.9%。新增省级重点龙头企业20家、富硒基地72个、国家地理标志保护产品5个。梅县区获国家农产品质量安全县称号。脱贫攻坚成效显著，全市精准减贫52306人。349个省定贫困村基本完成“三清三拆三整治”工作，新农村“四类示范工程”建设扎实推进，五华 “益塘流域·十里水乡”、平远河头“十里水乡·梦里老家”等连片示范点建设初见成效。建立全市地质灾害信息系统，完成266宗村村通自来水工程和400公里新农村公路路面硬底化，群众生产生活条件得到改善。深化农村土地制度改革，农村土地承包经营权确权登记颁证率达94.57%。梅江区、梅县区农信社完成申筹上报，梅州农商行筹建步伐加快。

五、社会各项事业有新进步。坚持教育优先发展，加快创建“广东省推进教育现代化先进市”，梅江区、蕉岭县创建成为粤东西北首批“广东省推进教育现代化先进县（区）”，完成24个教育强镇（街）复评。统筹发展学前教育和城乡义务教育，高中阶段教育水平进一步巩固提升，嘉应学院省市共建工作扎实推进。广泛开展群众文明创建活动，以全省最高分取得全国文明城市提名资格。传承客家优秀文化，客家文化（梅州）生态保护实验区总体规划获国家批准实施，新增省级非遗项目5项，第二批132处历史建筑颁证挂牌，加大江北历史文化街区保护开发。创作一批优秀文艺精品，29件作品获省以上奖励。梅州成为粤东西北地区阅读指数排名最高的地级市。加快创建卫生强市，城市公立医院综合改革全面铺开，市第二中医医院通过三甲评审，建立了一批紧密型医联体，基层医疗服务能力有效提升。全面两孩政策稳妥实施，出生人口素质不断提高。社区残疾人康园中心实现全覆盖。振兴“足球之乡”工作有新成效，成为目前全国唯一拥有两支中甲球队的城市。全民健身活动广泛开展，基本建成梅江南岸健身长廊。深入推进平安梅州、法治梅州建设，圆满完成党的十九大、第五届世界客商大会等重大安保维稳任务。我市群众安全感和政法工作群众满意度再获全省第一。加强社会综合治理，大力推进综治“中心+网格化+信息化”建设，有效化解社会矛盾。创新社区矫正、社区戒毒工作和安置帮教模式，在全省率先开展严重精神障碍患者监护人责任险投保工作。坚守安全红线不动摇，安全生产形势持续稳定。妇女儿童事业健康发展，国防动员、兵役征集、国防教育、双拥、国家安全等工作扎实推进，民族、宗教、外事侨务、港澳台和编制、统计、供销、粮食、气象、打私、打假、应急、人防、消防、档案、方志等工作取得新成绩。

六、民生福祉有新改善。坚持小财政办大民生，十件民生实事基本完成，民生投入占财政支出的八成。居民人均可支配收入19635元，增长9.2%。深化医保支付方式改革，完成机关事业单位养老保险改革，企业职工养老保险实现省级统筹，社保待遇水平全面提升。全市社会保险综合参保人数851.15万人次，增长5.41%。大力促进就业创业，全年城镇新增就业3.07万人、劳动力新增转移就业5.79万人次，促进创业2742人；城镇登记失业率2.45%。如期完成7809户农村危房改造，3791套（户）棚户区改造安置房开工建设。加强食品安全源头监管，全市117家农贸市场实现快检全覆盖。

七、政府自身建设有新提升。优化发展软环境，提升政务效能和水平。深化“放管服”改革，推行减证便民、审批便民和政府服务“马上办”，建成我市首个24小时网上办事自助服务区。梅州市政府公共服务总体满意度居粤东西北首位。坚持在法治轨道上开展工作，全面推进政务公开，加强基层政府网站集约化建设，政府民主科学决策水平不断提高。自觉接受人大及其常委会和政协的监督，建议提案按时办结率、代表委员满意和基本满意率达100%。进一步完善政府法律顾问机制，开展客家围龙屋保护、市容环境卫生管理、房屋使用安全管理的立法工作。开展市直机关作风整治，机关干部作风持续转变。突出专项治理，着力纠治扶贫领域腐败问题。加强重点领域、部门、项目和资金的审计监督。结合“两学一做”学习教育，深入开展党的十九大精神大学习、大培训、大调研。严肃党内政治生活，落实全面从严治党主体责任，严查干部不作为、慢作为或乱作为，营造良好的政治生态。

各位代表！过去的一年，我们克服了种种困难，取得了比预想要好的结果。这些成绩离不开省委、省政府的关心支持，离不开市委的正确领导，离不开全市上下的共同努力，也得益于广州市的鼎力帮扶。在此，我谨代表市政府，向全市人民、中央和省属驻梅单位、驻梅部队，向所有关心支持梅州发展的海外侨胞、港澳台同胞和社会各界朋友，表示衷心的感谢并致以崇高的敬意！

总结成绩的同时，我们也清醒地认识到当前面临的挑战和存在的问题，主要有：交通条件、营商环境比较优势欠缺，对市场主体吸引力不够强；产业发展不充分，县域经济发展不平衡，创新创业氛围不浓厚，推进供给侧结构性改革任务艰巨；有的干部不在状态、不敢担当，与新时期好干部的标准仍有差距，等等。对此，我们将高度重视，采取有效措施加以解决。

2018年工作安排

各位代表！2018年是站在新的历史起点上，全面贯彻落实党的十九大精神的开局之年，发展的机遇和动力前所未有。党的十九大描绘了新时代发展的宏伟蓝图，让人民群众对发展、创新、进步充满新的期待。省委、省政府更加重视解决区域发展、城乡发展不平衡不充分的问题，在补短板中释放短板潜力。省委十二届三次全会明确提出，要把握粤北山区生态发展定位，以生态优先和绿色发展为引领，加大财政转移支付力度，提高基本公共服务均等化水平，在更高水平上推动区域协调发展取得新突破，这为生态人文厚重的梅州注入新的发展动力。市委七届四次全会作出了践行新发展理念，推动高质量发展，坚持走生态富民强市新路子的部署，吹响了我市加快转型发展、绿色发展的新号角。我们要始终坚持发展第一要务，不断深化市情认识，把握好发展定位和发展方向，变发展机遇为发展动力，聚焦发展不充分的产业、尚未有效利用的资源，凝聚全市上下的力量，打好扬长和补短的组合拳，务实重干，精进前行，推动全市经济高质量发展，开创决胜全面建成小康社会新局面。

今年政府工作的总体要求是：以习近平新时代中国特色社会主义思想为指导，深入学习贯彻党的十九大和中央经济工作会议精神，以省委李希书记来梅调研讲话精神为动力，按照中央和省委、市委全会的部署，坚持稳中求进工作总基调，坚持新发展理念，紧扣社会主要矛盾变化，贯彻高质量发展的要求，以供给侧结构性改革为主线，统筹稳增长、促改革、调结构、惠民生、防风险各项工作，发展生态产业，壮大实体经济，推进改革开放，致力改善民生，加快构建“一区两带六组团”科学发展格局，推动生态富民强市。

今年的主要预期目标是：全市生产总值增长6.5%左右；固定资产投资增长20%左右，社会消费品零售总额增长9.5%，进出口总额增长1%；一般公共预算收入增长8%；居民消费价格指数控制在3%以内，居民人均可支配收入增长与经济增长基本同步；城镇登记失业率控制在3.5%以内；节能减排降碳约束性指标完成省下达年度计划。

实现今年的目标任务，重点是抓好以下工作：

一、持续扩大有效投资，为经济新发展夯实基础、创造条件

坚持不懈加大投资，既可以有效改善发展条件，又能形成新的经济增长点。今年将全力推进基础设施、产业和民生工程等省市重点项目建设，争取完成有效投资1000亿元以上。

再掀交通建设热潮。推进“两高一场”与国省道互联互通，加快构建便利、快捷的立体交通网络，确保大潮、梅平、东环、华陆、丰华高速无障碍施工；大力推进梅汕高铁建设，争取双龙高铁年内动工，继续做好瑞梅铁路前期工作；扎实推进梅县机场迁建和五华通用机场前期工作；实施普通公路基础设施重点项目建设三年行动计划，突出抓好国省干线和县乡公路升级改造，着力推进环城路等39个重点项目建设，加快未通客车行政村窄路拓宽和完成1400公里普通公路安全生命防护工程，努力建设“四好农村路”。确保梅畲快线十月底前建成通车。争取立项韩江三河坝至潮州港航道扩能升级工程。

推进水利能源等项目建设。坚持合理布局、适度超前，大力实施一批水利、能源、信息等项目。加快韩江高陂水利枢纽工程建设进度，建成船闸并具备通航条件；积极推进梅州抽水蓄能电站主体工程及其附属设施建设，争取益塘水库引水工程年内动工建设。继续推进山区中小河流治理，完成111.6公里年度治理目标。实施水土保持工程，全面完成“五沿”崩岗水土流失综合治理。加快推进500千伏变电站及配套输变电工程和产业集聚带配套电网建设。加大农村电网改造，年底前完成省定贫困村电网改造任务。完善信息基础设施，抓好726个行政村电信普遍服务试点建设，提升城乡信息化水平。

精心谋划和高效推进项目建设。汇聚政策、资金、土地等要素，保障项目顺利实施。认真研究国家和省的政策导向、投资方向、市场需求，聚焦生产性、基础性投资，超前谋划交通等重大项目，力争更多项目纳入国家和省的发展规划，获得中央、省预算内投资和省四大政策性基金支持。加强政银企合作，放大广东原苏区绿色发展投资基金效应，争取政策性优惠贷款向重点项目倾斜。鼓励发行企业债，补充项目资金来源，激发民间投资潜力。优化土地资源配置，完成省下达的年度垦造水田任务，实现占补平衡。加大闲置土地处置，鼓励租赁工业用地，扩大土地有效供给，提高土地开发投资强度和产出效率。

积极防范化解金融债务风险。全面清查地方政府债务、农信社等金融机构不良资产，充分预估预判房地产信贷、涉众金融等风险，切实做好重点领域风险防范和处置，把风险点逐一化解掉。规范PPP模式，清查变相举债等情况。探索创新省属国企与地方共赢发展模式，推动我市金雁集团、水务集团与广钢集团、建工集团深度合作，打造战略互投、地方控股的国有新平台，做强做优做大国有资本。

二、注重新旧动能协同发力，推动实体经济高质量发展

全市上下对实体经济的新认识，是加快产业发展的最大动力。我们要从供给端发力，实施创新驱动，加大科技、人才、品牌等要素投入，提升产业发展水平，努力构建现代化经济体系。

加快发展新型工业。坚持把广东梅兴华丰产业集聚带作为新型工业的主战场，加快水务环保、通用标准厂房等基础配套建设，推动科研机构、教育、医疗等资源向园区积聚。推进创新创业孵化园、专利技术孵化产业园、广铁物流园等配套平台建设，争取国家级高新区、综合保税区申报工作有突破性进展，力争梅州经济技术开发区升格为国家级，力促公共技术服务平台和创新创业服务体系建设上新水平。高质量推进穗梅产业共建，以广梅园为核心、各县（市、区）产业园为支撑，力争引进20个亿元以上项目落户园区。拓宽“总部+基地”共建模式，积极承接珠三角产业梯度转移，努力打造跨区域产业链。

改造提升传统产业。全面落实省“实体经济十条”和创新驱动“八大抓手”，成立技改投资扶持基金，支持企业增资扩产、技术改造。加快产品结构调整，推动烟草、建材、机电、陶瓷等传统产业向中高端发展。促进制造业提质升级，巩固高端电路板优势产业，做精做优电声产业，做大做强自行车产业。开展质量提升行动，引导企业提品质、创品牌。推动工业设计工作，培育发展国家、省级工业设计中心。加大力度扶持中小企业发展，实施重点企业规模与效益倍增计划，切实做好“育上规”、“保规上”、“个转企”。灵活运用并购重组等手段，做大现有上市企业，力推2家企业申报国内主板上市。

大力发展现代服务业。坚持文旅、农旅深度融合，加快梅江韩江绿色健康文化旅游产业带建设。实施“大景区”整体规划，丰富旅游产品，延长旅游产业链条，推动“一处美”向“一片美”升级。争取成立旅游投资基金，完善旅游基础配套建设，力争年内创建成为国家全域旅游示范市。加大红色资源的保护利用和开发，充分挖掘红色文化的历史内涵，融合生态旅游、乡村旅游、会展经济等功能，把“八一”起义军三河坝战役纪念园、叶剑英纪念园、梅南九龙嶂革命纪念馆打造成为传承红色基因、发展红色旅游的示范区。加强区域旅游合作，整合旅游资源和特色旅游线路，打造粤闽赣客属地区无障碍旅游区。推动传统企业与电商融合发展，加快物流园智能化和跨境电商产业园建设，规范快递行业监管。推进4K电视网络应用与影视等产业发展，促进信息领域的消费升级。积极发展新能源、新材料和生物医药等新兴产业，推动养老养生、健康休闲、体育健身等新业态加速发展。

发展壮大县域经济。立足县域实际，做好各县（市、区）特色产业发展规划，加速培育1-2个特色鲜明、辐射带动力强的主导产业。坚持产城联动，结合县级新区开发，推动各县（市、区）工业园区和集聚地建设，打造独具特色的新型产业基地，增强县城、中心镇的集聚能力，支撑县域经济发展。全面落实县域经济社会发展扶持政策，赋予县级更大自主权，增强基层的工作主动性和创造性。

三、加快新型城镇化建设，促进城乡融合发展

顺应新的发展要求，重塑新型城乡关系，既要提升城市发展水平，又要探索建设特色小镇等新型发展平台。

加快中心城区建设。立足长远，加强对城市规划、公共服务、城市经济的系统研究，提高城市发展的持续性、宜居性。统筹推进嘉应新区起步区建设，全面完成江南新城、芹洋半岛5个安置区建设任务。按照海绵城市和国家地下综合管廊试点城市标准，加快推进道路和地下综合管廊等配套设施建设，年底前基本建成“两馆两场一园”和三角跨线桥等市政路网工程。动工建设市旅游服务中心等公共服务项目，不断完善功能配套，优化提升新城环境。坚持产城融合，加快推进嘉应新区起步区40个产业项目建设；改造提升江北片区，以文旅、医康养、商贸物流为发展重点，激发经济增长新动力。

大力发展特色小镇。坚持高起点规划，抓好《县（市）域乡村建设规划》编制，统筹城镇空间和基础设施布局，增强公共服务供给。立足资源禀赋，注重生产、生活、生态的融合，突出产业建镇，结合广梅园、东升园等园区建设，结合电声、陶瓷、文化旅游等专业镇和中心镇“六个一”发展，开拓产业发展路径，建设一批绿色工业、生态文化旅游、精致农业和足球等特色小镇。突出品牌打造，结合美丽乡村和山水田园综合体建设，集中优势资源，重点推进留隍镇国家新型城镇化综合试点和东山健康小镇、雁洋文化旅游小镇等省级示范点建设，塑造精品、提升品位。突出功能配套，按照适度超前、集约利用的原则，加强特色小镇的基础设施和公共服务建设，营造良好的环境，吸引产业、资本和人才，打造创新创业和享受生活的美好家园。

推进城乡环境综合治理。坚持建管并重，提升城市形象。深化城市管理执法体制改革，进一步理顺市与区、县的管理职能，促进城市管理向治理转变。加强城乡街道和社区服务管理，实施城市净化绿化亮化美化工程。强化背街小巷环境整治，开展泥头车、小广告、空调主机占道、架空管线等专项治理，大力整治乱摆乱卖乱建、深夜扰民等行为；建设多样性、功能性、艺术性的城市绿地；提升城市景观照明，点亮城市天际线；打通客都大桥东端连接线、新峰桥至205国道等路段，在人流车流繁忙路段建设人行天桥，改善城市“微循环”，打造整洁、有序、畅通的市容环境。加快智慧城市建设，完善市县两级数字化城管平台，实现精细化、网格化管理。提高镇村规划水平，优化村庄布局，进一步规范农村建房。加大村容村貌整治力度，对不同风貌进行分类指导，确保年底完成90%的自然村环境综合整治任务。

四、实施乡村振兴战略，激活农村发展力量

梅州作为传统农业大市，发挥农业比较优势，推动乡村振兴，有着巨大的潜力和后劲。

推动农村一二三产业融合发展。坚持质量兴农、绿色兴农，顺应消费主流的需求，推广良种良法和生态种养，发展高端生态农业，推动农业生产规模化、林下经济特色化发展，努力打造优质农产品生产供应基地，让梅州农产品成为绿色产品代名词。大力引进、培育新型农业经营主体，借助先进理念和技术，发展农副产品精深加工，提升农产品附加值。发挥农业龙头企业的带动作用，推进农业产业化经营，打造从“田间到舌尖”的产业链条，今年新增10家以上省级农业龙头企业。积极发展农村电子商务，完善农产品营销服务网络，加快建设农产品专业交易市场，完善仓储、物流冷链配套设施，推动农副产品走向更广阔的市场。培育农村新业态，重点发展农业+旅游、农业+体验、农业+文化、农业+康养等产业；引导和支持社会资本，开发农民参与度高、受益面广的休闲旅游项目，带动乡村旅游蓬勃发展。用好金融惠农支农政策，推动农业企业登陆新三板、在区域性股权交易市场挂牌，增强直接融资能力。引导农业专业技术人才、农产品贸易能手回乡创业，带动培养一批有文化、懂技术、会经营的新型农民。

深化农村改革。推进农村土地制度和集体产权制度改革，落实农村土地承包经营权再延长30年的政策，全面完成土地确权颁证任务。推行农村“三变”改革，确保农民从土地流转中获取收益。健全农业社会化服务体系，实现小农户和现代农业发展有机衔接。建立健全城乡融合发展体制机制，清除阻碍要素下乡的各种障碍，加快公共交通、供水、垃圾处理、消防等基础设施向农村延伸，推动教育、卫生、文化等公共资源向农村倾斜。

推动乡村善治。创新乡村治理体系，发挥群众主体作用，推动农村自治、法治和德治相结合。完善民事民治管理机制，发挥村民理事会等社会组织作用，广泛发动乡村制订村规民约，引领农村新风尚。深入开展农村法治宣传教育，引导农民学法守法用法，增强法治意识和依法维权能力。大力宣传和弘扬社会主义核心价值观，开展文明镇村评比、立家训家规等活动，厚培文明乡风。

坚决打赢脱贫攻坚战。围绕“三年攻坚、两年巩固”要求，切实履行主体责任、属地责任，注重扶贫同扶志、扶智相结合，保证脱贫质量。全面落实产业、就业和金融等帮扶措施，拓宽贫困户增收渠道。加强实用技术培训，促进贫困人口就业，鼓励有条件的农民自主创业，带动就业。抓好政策性扶贫，实现应保尽保，确保不因贫辍学、防止因病致贫。管好用好专项扶贫资金，提高精准扶贫成效，努力实现“小康路上一个都不能掉队”。

五、厚植绿色优势，保障生态产业发展

绿水青山就是金山银山，也是梅州人民的幸福靠山。保护好环境，是推进生态富民强市的重要前提。

强化生态环境治理。围绕打造广东林海，持续开展“绿满梅州”大行动，抓好高速公路沿线、旅游干线第一重山的造林绿化，完成20.247万亩林相改造和省级乡村绿化美化任务。加强自然保护区、森林公园、湿地公园等管护，加快国家森林城市、广东省森林小镇创建工作，推进市植物园建设。落实森林火源管理条例，严防森林火灾。强化大气污染防治及工地道路扬尘污染、工业源污染、露天焚烧污染、油烟排放等防控，建成使用城区垃圾焚烧发电厂，确保城区空气质量优良率达到96%以上。全面开展重点行业企业用地土壤环境质量调查，推进土壤污染管控和修复，加强农业面源污染防治，保障人居环境健康和农产品安全。全面推行河长制，加大力度整治黄塘河、周溪河黑臭水体，推动江河湖库治理长效化常态化；强化河流交接断面水质保护和考核，确保达标率100%，实现一江清水向东流。

推动绿色低碳发展。加快创建国家环保模范城市，全面完成节能减排任务。推进生产方式绿色化，重塑绿色能源结构。加强工业园区循环化改造，因地制宜发展绿色产业、环保产业。以建设绿色交通示范市为契机，发展绿色公交，实现城区公共汽车100%纯电动化；加强城市绿道等公共慢行系统建设，鼓励全民低碳出行。推进生活方式绿色化，开展创建节约型机关、绿色学校、绿色社区、绿色家庭等行动，建立绿色消费机制，减少一次性产品消费。加强生态文明宣传教育，强化群众环境意识，推动全社会形成绿色发展的良好风尚。

建立健全生态文明制度。推进国家生态文明示范区建设，抓好生态文明建设体制机制创新，完善绿色发展指标体系，严格执行省主体功能区产业准入负面清单。实行国土空间开发保护制度，严厉打击破坏耕地、水田建房、非法买卖土地等行为。严格落实森林、绿地、河湖水系等生态保护红线管控，加强自然生态空间保护。认真落实领导干部任期生态文明建设责任制。实行最严格的环境保护监管制度，建立健全环保责任追究、生态补偿和资源有偿使用等制度。加大环境执法力度，全面完成中央环保督察反馈问题整改任务。

六、深化改革创新，构建对外开放新格局

改革开放是推动发展的“关键一招”。要以改革开放40周年为新起点，坚持开放促发展，改革增活力，推动梅州更好地融入开放共赢的时代。

深化重点领域改革。严格按照顶层设计，紧紧抓住事关全局的重大改革，充分释放经济社会发展活力。加大力度推进“去降补”，加快淘汰落后产能，着力推进降低制度性交易成本系列改革，补齐基础设施短板。加强房地产调控，确保房地产市场稳定健康发展。深化“放管服”改革，完善“一门式一网式”政府服务模式改革，全链条、全流程优化办事环节；深化营商环境、商事制度和投融资体制改革，完善“多证合一”改革，推进“双随机一公开”监管，加快简政放权，提高行政效率。加快金融体制改革，年内完成农信社改制工作。推进财政管理体制改革，促进市与县（市、区）财权与事权相匹配。理顺市与区国土资源管理体制，依法规范土地管理。

扩大对外交流合作。积极参与“一带一路”建设，探索建立与境外投促机构、商协会的合作机制，争取在欧洲、非洲增设1-2个海外经贸文化联络处。积极创建国家级一类航空口岸，扩大对外开放。发挥世界客商大会平台效应，落实赣闽粤原中央苏区合作框架协议，加强与海西区、赣南等地的经济互动、产业合作，推动协同发展。推进大埔陶瓷、丰顺电声申报国家级外贸转型基地，培育发展电子信息、家具、工艺品等特色外贸基地，拓展国际市场。培植外贸新增长点，新备案60家以上对外贸易经营者登记企业。坚持引资引智引技并举，善于借力发展，争取更多的优质项目落户，吸引更多的优秀乡贤回归。

推进创新创业。集聚创新要素，弘扬工匠精神，推动技术创新、产业发展。认真实施高新技术企业树标提质计划，争取新认定20家高新技术企业。推进产学研深度融合，增加R &D经费投入，引导规上企业建立研发机构，组建1家以上国家级科技企业孵化器，争取年内分别建成1-2家新型研发机构、院士工作站。推进世界客属青年创业创新中心和院士驿站等平台建设，引进高端技术团队进驻。加强知识产权保护运用，促进专利技术成果转化。发挥中小微企业信贷风险补偿基金、促进创业小额担保贷款基金等作用，推广应用创业券创新券，激励中小微企业创新发展。

七、坚持以人民为中心，持续改善社会民生

民生是为政之要。切实解决好人民最关心最直接最现实的利益问题，让发展更有温度、幸福更有质感。

办好十件民生实事。一是提高困难群众救助补助标准。城镇、农村低保对象最低生活保障人均补差水平，分别从每月457元、206元提高到503元、228元。特困人员基本生活标准按不低于当地最低生活保障标准的1.6倍、且不低于当地现行特困人员基本生活标准确定。孤儿基本生活最低养育标准集中供养和分散供养水平，分别从每人每月1450元和880元提高到1560元和950元。城乡低保对象政策范围内基本医疗救助比例达到80%以上，建档立卡贫困人口政策范围内基本医疗救助比例达到80%以上。困难残疾人生活补贴、重度残疾人护理补贴标准，分别从每人每年1800元、2400元提高到1890元、2520元。二是扶持就业创业。促进创业2500人、城镇新增就业2.5万人、失业人员再就业2.3万人。对符合条件参加职业技能晋升培训、法定劳动年龄内的省内外城乡劳动者按规定给予补贴，提升1万人次就业技能水平。实施“圆梦计划”，资助100名在梅务工的优秀新生代产业工人参加学历继续教育的专、本科学习。三是提高边远地区学校教师生活补助。鼓励引导人才向边远贫困地区流动并长期从教，将农村边远地区学校教师生活补助从每月人均不低于900元提高到不低于1000元。四是改善困难群众居住条件。开工建设棚户区改造安置房1380套（户）、基本建成4914套，发放租赁补贴380户。全面完成省下达的农村危房改造任务。五是提升医疗卫生养老保障水平。将职工基本医疗保险基金年度累计最高支付限额从9万元提高至10万元，职工补充医疗保险年度累计最高支付限额从27万元提高至28万元。将职工医疗保险市内住院起付线一级医院从450元降低至200元、二级医院从600元降低至450元、三级医院从800元降低至650元；将城乡居民医疗保险市内住院起付线二级医院从500元降低至450元、三级医院从800元降低至650元。将城乡居民大病保险起付标准从10000元降低至8000元、报销比例从70%提高至75%；城乡五保供养对象、建档立卡贫困人员起付标准从2000元降低至1000元，报销比例从80%提高至85%，不设年度最高支付限额。城乡居民基本养老保险基础养老金最低标准从每人每月120元提高至138元。升级改造5家中心卫生院、9家县级人民医院、7家县级中医院以及6家县级妇幼保健院，其中5家中心卫生院基本完成主体基建工程。扩大家庭医生签约服务人群，实现家庭医生签约服务覆盖率达30%以上、重点人群达60%以上。六是增加教育体育公共资源供给。镇中心以上学校全部实施校园网提速工程，实现光纤网络延伸入班，普通教室配备多媒体教学设备；教学点实现30%以上。建设城乡15分钟健身圈，为省定贫困村配建一套8件以上器材的健身路径。完成1个、动工9个公共体育场馆建设。七是巩固提升人居环境。全市349条省定贫困村基本建成新农村整洁村。实施355宗村村通自来水工程建设。完成削坡建房搬迁治理1383处。改造梅城梅水南路、三板桥路、南堤（梅州大桥至嘉应大桥段）堤顶路面排水系统。安装梅城老城区550个市政消防栓。建设梅州市生态环境自动监测网络、梅州市树木园和30个以上瞭望台、300个森林消防蓄水池。八是实施交通便民工程。完成国省道新改建及路面改造125.19公里，改造国省道危桥19座，整治急弯陡坡、临水临崖等影响安全通行路段641公里。城区新增236辆纯电动公交车，投放2000辆公共租赁自行车、新建公共自行车驿站120座和系统管理中心。改造城区梅江大道、江南路和广梅路沿线48座公交站亭。九是健全社会矛盾纠纷化解机制。支持镇（街道）人民调解委员会聘请专职人民调解员，实行“以案定补”制度，加强调解组织规范化建设，调解成功率不低于97.5%。十是增加农民农业生产补助。对拥有耕地承包权的种地农民（含农场职工），按平均每亩不低于85元进行直接补贴，补贴总面积不少于150万亩。增加政策性农业保险保费补贴险种、扩大保险覆盖面、提高保障标准，其中水稻保险参保率达到80%以上，每亩每造保额从400元提高到800元。对全市1031.61万亩省级以上生态公益林，按规定提高每亩补偿标准。

加快教育现代化建设。全面推进创建“广东省推进教育现代化先进市”，力争年底实现县（市、区）全覆盖。坚持合理布局，落实小区配建学校政策。扩大公办幼儿园比例，各县（市、区）城区新建1所公办幼儿园；积极推动普惠性民办幼儿园建设。推进市实验小学、芹洋学校等一批市区和县城中小学校建设，提供更多优质学位，逐步解决大班额问题。督促学前教育依法办园，规范学生校外托管和培训机构管理。稳步推进营养改善计划，争取早日实现农村义务教育学生全覆盖。走质量强校之路，以10所省国家级示范性高中建设为重点，推动普高教学质量和办学水平不断提升。深化产教融合、校企合作，推进广梅共建职业教育，设立“现代学徒制”大专班，加快梅州南方紫琳职业学院筹建工作。深入开展省市共建嘉应学院，推进政产学研合作，提升服务地方能力。推进“三通两平台”建设，提升教育信息化水平。加强师德师风建设，推进中小学校长职级制改革和基础教育学校公办教师“县管校聘”改革。重视特殊教育，完善贫困残疾学生特殊学习用品、教育训练等补助政策，促进残疾儿童享有平等教育权利。鼓励社会力量和民间资本参与办学。支持开放教育发展，为全民学习、终身学习提供公共服务。

协调发展文化等各项事业。扎实创建全国文明城市，深化城乡“四级联创”。大力弘扬优秀客家文化和红色文化，打造广东文化名城。推进客家文化（梅州）生态保护实验区建设，加强客家围龙屋保护，推进自然村落历史人文普查，活化利用桂芳楼、七贤居和镇东楼片区等传统古民居。筹建中央苏区（广东）历史纪念馆和广东汉剧博物馆。建成2129个基层综合性文化服务中心，实现行政村全覆盖。培育文艺精英人才，打造精品力作，繁荣客家文艺，继续办好客家文博会。推进《振兴足球三年行动计划》，打造足球城市品牌。抓好青少年体育工作，争取第十五届省运会获得好成绩。积极筹备第二批海峡两岸交流基地示范点申报工作。落实粮食安全政府责任制，确保粮食安全。加强国防动员工作，推动军民融合创新发展。进一步做好国家安全、人防、双拥、外事侨务、气象、防灾减灾、民族、宗教、统计、方志、档案、打私、打假、应急等各项工作。

加强社会保障。加快创建卫生强市，建设健康梅州。推进“三医联动”，落实分级诊疗制度，加快推进医联体建设。市人民医院加快创建省粤东西北区域医疗中心，完成粤东医院创三甲工作。加强基层全科医生队伍建设，提升医疗保障服务能力。大力推进国家卫生县城创建，确保顺利通过国家卫生城市复审。完善全面两孩相关配套政策和制度。实施全民参保计划，健全社会保险制度体系。落实党政同责，强化食品药品源头治理，保障群众舌尖上的安全。注重医养结合，推进社区居家养老，促进和规范养老机构发展。加大对残疾人精准康复服务和就业创业的扶持力度，对贫困重度残疾人家庭进行无障碍改造。维护妇女儿童合法权益，健全留守儿童、困境儿童关爱服务体系，从源头上减少农村留守儿童。

强化社会治理创新。加快民主法治创建，完善“七五”普法考评指导标准和指标体系。激活社会组织活力，加强志愿者服务工作，提升社区治理法治化、科学化、精细化水平和组织化程度。做好社区矫正工作，探索社区戒毒社区康复多元模式，深入推进全民禁毒工程。完善公平竞争审查制度，强化社会信用体系和市场监管体系建设，维护市场经济秩序。树立安全发展理念，全面压实安全生产责任，强化重点行业领域安全生产攻坚。严厉打击各类违法犯罪，健全信息化、立体化社会治安防控体系，推进“智慧公安”、“平安梅州”视频建设，加强公安派出所基层基础建设，树立移动警务车品牌，进一步提高路面见警率、管事率；加强网络安全建设，着力解决网上虚假信息诈骗、倒卖个人信息等问题，提升公众安全感和满意度，建设更高水平的平安梅州、法治梅州。

各位代表！新的发展形势，对政府工作提出了更高的要求。我们将高举习近平新时代中国特色社会主义思想伟大旗帜，认真学习贯彻落实党的十九大精神，深入开展“不忘初心、牢记使命”主题教育，持之以恒加强政府自身建设，增强政府公信力和执行力。坚持依法行政，完善依法决策机制。自觉接受市人大及其常委会的依法监督，支持市政协开展各项履职活动，主动听取民主党派、工商联、无党派人士和人民团体意见建议，办好人大代表议案、建议和政协委员提案。坚持科学立法、民主立法，提高政府立法公众参与度。加强政府作风建设，始终把规矩和纪律挺在前面，严格贯彻落实中央八项规定和实施细则精神，驰而不息纠治“四风”，治理庸政懒政怠政。全面构建亲、清新型政商关系，真心实意为投资者提供服务。加大审计力度，保障经济运行安全。强化扶贫等重点领域监督执纪问责，无禁区全覆盖零容忍推进反腐败斗争。加强公务员诚信教育和管理，以政府诚信引领提升全社会诚信水平。整合政务信息系统，加快“数字政府”建设，推动公共资源配置领域政府信息公开、全程留痕，提升网络问政平台服务能力，依法及时就地解决群众合理诉求。大力弘扬“马上就办”、雷厉风行的作风，对部署的工作抓紧快办，定一件做一件，办一件成一件，树立政府务实为民的良好形象。

各位代表！幸福是奋斗出来的。新的起点，目标在前，重任在肩。让我们更加紧密团结在以习近平同志为核心的党中央周围，在市委的坚强领导下，一步一个脚印，踏踏实实干好工作，加快建设“一区两带六组团”，推动生态富民强市，为决胜全面建成小康社会而努力奋斗！
